# M. Piorry's New Stethoscope

**Published:** 1830-10-01

**Authors:** 


### LIII.

### M. PIORRY'S NEW STETHOSCOPE.

M. PIORRY sets out with the position, that metals will conduct sound sufficiently well to admit of being employed as stethoscopes. Metal will also serve for the pleximeter, or that thin plate attached to the large end of the modern stethoscopes, generally made of ivory, and used for percussion. Having ascertained these facts M. Piorry determined to apply them to the construction of an instrument, which should be at the same time light and portable. His new stethoscope consists of a tube of copper two lines in diameter and six inches long; the length admitting of being increased to the employer's taste, by fixing a second tube upon the first. The pleximeter should be eight lines in diameter, and of copper also, well polished, rounded and fitting well at the edges. Two lines from a point of its circumference is an opening which screws on one of the ends of the metallic tube. The *opercule* is an inch and a half in breadth, and in its centre is a hole which receives the other end of the cylinder. If the impulsion of the heart, pectoriloquy, bronchophony, or ægophony are to be explored, the flat side of the pleximeter is to be applied to the chest; for the respiration, sounds of the heart, and so forth, we must employ the hollow side. M. Piorry imagines that the great portability of this instrument will render mediate auscultation more generally available than it is at present. For our own parts we think the wooden stethoscopes now in use are by no means incommodious. The shaft is extremely slim, and consists of two separable portions, whilst the ear-piece and ivory pleximeter screw off the stethoscope and fit upon each other.

